# A Case of XX Disorder of Sexual Development in a Female-Phenotype Roe Deer (*Capreolus capreolus* L.) Associated with Antlers Growth with Retained Velvet

**DOI:** 10.3390/ani12070865

**Published:** 2022-03-29

**Authors:** Filipe Silva, Isabel Pires, Justina Prada, Miguel Queirós, Aurora Monzón, José Almeida, Roberto Sargo, Filipa Loureiro, Luís Sousa, Joana Valente, Carlos Viegas, Mário Ginja, Estela Bastos, Ana Martins-Bessa, Isabel Dias

**Affiliations:** 1 Exotic and Wild Animal Service, Veterinary Hospital of University of Trás-os-Montes e Alto Douro (UTAD), P.O. Box 1013, 5000-801 Vila Real, Portugal; fsilva@utad.pt (F.S.); jprada@utad.pt (J.P.); roberto.sargo@gmail.com (R.S.); filipaloureiro@gmail.com (F.L.); luissousa@utad.pt (L.S.); joanvalente@gmail.com (J.V.); cviegas@utad.pt (C.V.); mginja@utad.pt (M.G.); idias@utad.pt (I.D.); 2 Department of Veterinary Sciences, School of Agrarian and Veterinary Sciences, University of Trás-os-Montes e Alto Douro (UTAD), P.O. Box 1013, 5000-801 Vila Real, Portugal; ipires@utad.pt (I.P.); malmeida@utad.pt (J.A.); 3 Veterinary and Animal Science Research Centre (CECAV), University of Trás-os-Montes e Alto Douro (UTAD), P.O. Box 1013, 5000-801 Vila Real, Portugal; 4 Department of Genetics and Biotechnology, School of Life and Environmental Sciences, University of Trás-os-Montes e Alto Douro (UTAD), P.O. Box 1013, 5000-801 Vila Real, Portugal; miguelqueiros.95@gmail.com (M.Q.); ebastos@utad.pt (E.B.); 5 Centre for the Research and Technology of Agro-Environmental and Biological Sciences (CITAB), University of Trás-os-Montes e Alto Douro (UTAD), P.O. Box 1013, 5000-801 Vila Real, Portugal; amonzon@utad.pt; 6 Department of Forest and Landscape, School of Agrarian and Veterinary Sciences, CIFAP, University of Trás-os-Montes e Alto Douro (UTAD), P.O. Box 1013, 5000-801 Vila Real, Portugal

**Keywords:** roe deer, disorders of sexual development, DSD, ovotestis, antler growth, velvet, *SRY*, PIS

## Abstract

**Simple Summary:**

Disorders of sexual development (DSDs) are characterized by incongruity between the genetic and phenotypic sex, which can be caused by anomalies in sex differentiation or gonad sex development, or abnormal sexual-hormone secretion and receptor-expression patterns during embryonic development. DSD cases can retain both ovarian and testicular tissues in one single gonad (ovotestis) or in two separate gonads. These disorders have already been described in different mammalian species, including domestic animal species. However, reports of sexual abnormalities in wild species are scarce. The present work reports a specimen of roe deer (*Capreolus capreolus* L.) with an intersex phenotype, as the animal presented antler growth in the presence of a typically female genital phenotype. A clinical, histopathological, and molecular study was subsequently performed.

**Abstract:**

A 3-to-4-year-old roe deer (*Capreolus capreolus* L.) was admitted to the Veterinary Hospital. Although it showed well-developed antlers with retained velvet, an external female appearance and genitalia were evident. External biometrical measurements were taken for the antlers, and a computed tomography was performed. Molecular studies targeting the *SRY* gene were performed, and a PIS (polled intersex syndrome) mutation diagnosis was implemented. The gonads consisted of a right testicle paired with a left ovotestis. Histologically, the ovary-like structures in the ovotestis were functional, but the testis, as the testis-like structure in the ovotestis, did not show active spermatogenesis. No evidence of *SRY* gene was detected by PCR, suggesting an XX-chromosome constitution. Additionally, polled intersex syndrome (PIS) deletion was not detected in the case under study. The clinical and histopathological findings confirmed the DSD with the presence of a testicle and a contralateral ovotestis.

## 1. Introduction

Roe deer (*Capreolus capreolus* L.) have low sexual dimorphism, with males showing deciduous antlers, although occasionally, females can also develop antlers [1,2]. In male deer, antlers grow under androgen influence, starting with a rapid growth stage with antlers covered with highly vascular velvet, proceeding to a second stage involving the interruption of the blood supply, a stage in which there is the shedding of the velvet, thus exposing the hard horn, and ending with a final stage in which the antlers are cast off [3]. Retained velvet, sometimes combined with abnormal antler formation, is mostly observed in castrated bucks, but it has also been reported in wild animals [4]. In female roe deer, antlers with retained velvet are usually associated with older age. Stimulation with male hormones does not produce a sufficiently high testosterone peak for the shedding of the velvet [2]. 

Disorders of sex development (DSDs), formerly classified as true hermaphroditism and pseudohermaphroditism, are important conditions affecting reproduction. DSDs are characterized by differences between the genetic and phenotypic sex caused by anomalies in sex differentiation and gonad development, or due to abnormal sexual-hormone secretion and receptor-expression patterns during embryonic development [5]. Individuals can retain both ovarian and testicular tissues, in one single gonad (ovotestis) or in two separate gonads. These disorders have already been described in different mammalian species, including domestic animal species [6,7]. In wildlife species, such as roe deer, reports of sexual abnormalities are still scarce. The partial or complete reduction in fertility that results from DSD conditions is detrimental to the reproductive potential of wildlife populations and, consequently, their long-term survival [8]. Pajares et al. [9] described an *SRY*-negative XX case, the first description of abnormal genital development in an Iberian roe deer. Kropatsch et al. [10] described a DSD phenotype resulting from incomplete male determination in roe deer.

A complex network of regulatory genes controls sexual development in mammals. The *SRY* (sex-determining region on the Y chromosome) gene and its expression are considered necessary and sufficient to drive testis development [5]. Additionally, *Sox9* is, similarly to *SRY*, necessary and sufficient to initiate testis development. Apart from the mentioned genes, other regulatory genes are integrated in the network that controls sexual development in mammals. Recent reviews present a detailed overview of this network [11]. Simultaneously, the role of epigenetics in mammalian sex determination has been widely discussed in recent years, and is described in detail in the review of Miyawaki and Tachibana [12].

Polled intersex syndrome (PIS) is a cause of DSDs in goats [13]. Pailhoux et al. [14] demonstrated that the mutation underlying PIS is the deletion of an 11.7 kb DNA sequence. This deletion affects the transcription of two genes: PIS regulated transcript 1 (PISRT1), encoding a 1.5-kb mRNA devoid of an open reading frame (ORF); and forkhead transcription factor gene (FOXL2). Recent work by Simon et al. [15] revealed new genomic features of the polled intersex syndrome variant.

The aim of the present work was to report a case of DSD in a roe deer (*Capreolus capreolus* L.) that presented male features, with antler growth, showing retained velvet, and a female genital phenotype. Clinical, CT, histopathological, and molecular studies were carried out. 

## 2. Materials and Methods

### 2.1. Animal

**Figure 1:**
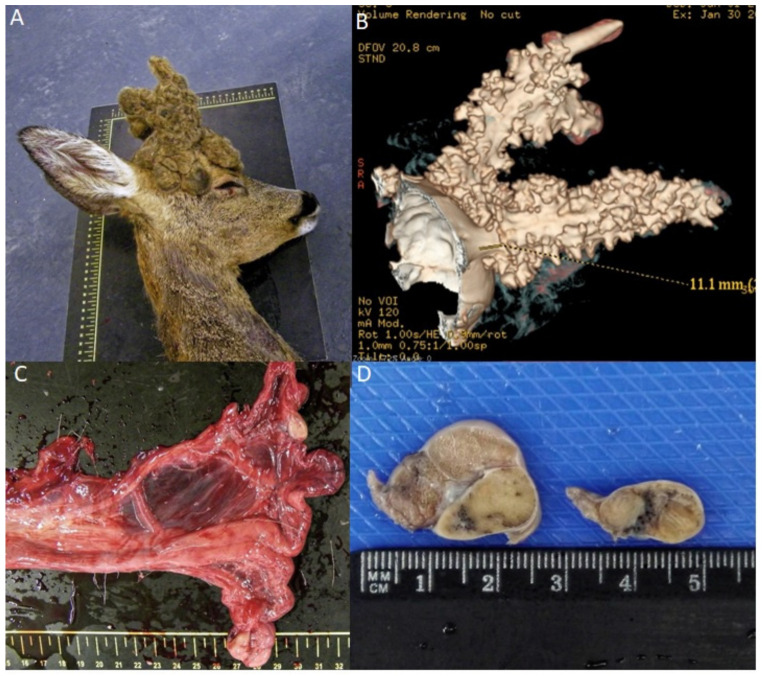
(**A**) Image of the antlers showing retained velvet; (**B**) CT image of the antlers; (**C**) Macroscopic view of reproductive organs, with two ovoid unilateral gonads; (**D**) the testicle (gonad at left), and the ovotestis (gonad at right), which showed two macroscopic distinct areas.

A roe deer from Northern Portugal was presented to the Veterinary Teaching Hospital after being hit by a car. Despite immediate treatment, the animal died from internal injuries and damage to the central nervous system. The animal exhibited an external female phenotype, accompanied by typical male antler formation with retained velvet (Figure 1A). 

The roe deer was weighed and examined biometrically (body length, shoulder height, ear length, and length of hind foot) using a tape measure at a scale of 1 mm. The age was estimated from the tooth wear (principally PM_3_ and M_1_) [16]. 

### 2.2. Computed Tomography Study

A computed tomography (CT) scan of the head and antler region was performed (CT Brivo CT325, General Electric^®^ Portuguesa, Carnaxide, Portugal, 1 mm-thick sections with helicoidal acquisition).

### 2.3. Molecular Study

A muscle sample was obtained from the cadaver under study. Simultaneously, blood samples were obtained from four healthy roe deer—two males and two females—in order to make controls. DNA was extracted from blood and muscle tissue using the QIAamp^®^ DNA Mini Kit (Qiagen, Hilden, Germany) following the manufacturer’s instructions. In the last step, two elutions, both using 100 μL of Buffer AE, were carried out. Two different approaches were applied to these DNA samples: (a) the presence of the *SRY* gene was tested using a protocol developed by Meadows et al. [17] for ovine; and (b) an amplification test was carried out following the protocol of Monteagudo et al. [13] for the diagnosis of PIS (polled intersex syndrome) mutations in goats (Table 1). 

The PCRs were performed in 15 µL volumes, using the DreamTaq PCR Master Mix kit (Fermentas, Thermo Fisher Scientific, USA) in a reaction mixture containing 7.5 µL of DreamTaq PCR Master Mix (2x), 1 µL of each primer (16 pmol), 1 µL of genomic DNA (100 ng), and 9.5 µL of H_2_O. The amplification reactions were performed with an initial denaturation at 95 °C for 3 min, followed by 40 cycles of 95 °C for 30 s, 55 °C for 30 s (the annealing temperature for the PIS fragment; in the case of the *SRY* fragment, the temperature was 50 °C), 72 °C for 30 s, and a final extension at 72 °C for 10 min. These PCR parameters were used in all of the reactions. Agarose gel electrophoresis was used to separate the PCR fragments. The *SRY* amplification product was purified using the ExoProstar™ 1-Step Illustra™ kit (US77702, Cytiva, Cardiff, UK) and sequenced in both directions.

### 2.4. Histopathological Study

A systematic postmortem examination was conducted within 3 h of death, with a collection of samples from various tissues, namely, the reproductive system, taken for histopathological analysis. The specimens were fixed in 10% neutral-buffered formaldehyde. The tissues were embedded in paraffin wax, sectioned at 3 µm, and stained with hematoxylin and eosin (H&E).

## 3. Results

### 3.1. Age Determination and Clinical Evaluation

The biometric measurements of the animal were as follows: total length, 123 cm; shoulder height, 72 cm; ear length, 12 cm; and length of the hind foot, 29.5 cm. The age was estimated to be between three and four years old according to the presence of a two cusp third pre-molar, with M_1_ still high and sharp. In this last tooth, the dentine had a half-moon shape in both cusps (antero- and posterolingual) and infundibulum. On external examination, the animal was considered an adult female, weighing 20 kg, with female external genitalia including vulvae and four mammary glands. There was no external evidence of testicles or a penis. 

### 3.2. CT Study

The CT scan (Figure 1B) revealed that the bone antler consisted of pedicles, two main beams and tines. The length of the left main beam was 111 mm without tines, and the length of the right was 116.9 mm, with two tines in the posterior position. The longer tine was approximately 41.4 mm, and the smaller tine was 12.2 mm in length.

### 3.3. Histopathological Study

An internal macroscopic examination revealed a complete female reproductive tract with a vagina, a cervix, and two uterine horns (Figure 1C). Bilateral oval gonadal structures were observed at the proximal end of the uterine horns, the right measuring 2 × 1.7 cm, and the left, 1.5 × 1 cm. The left gonad showed two distinct macroscopic areas (Figure 1D). 

**Figure 2:**
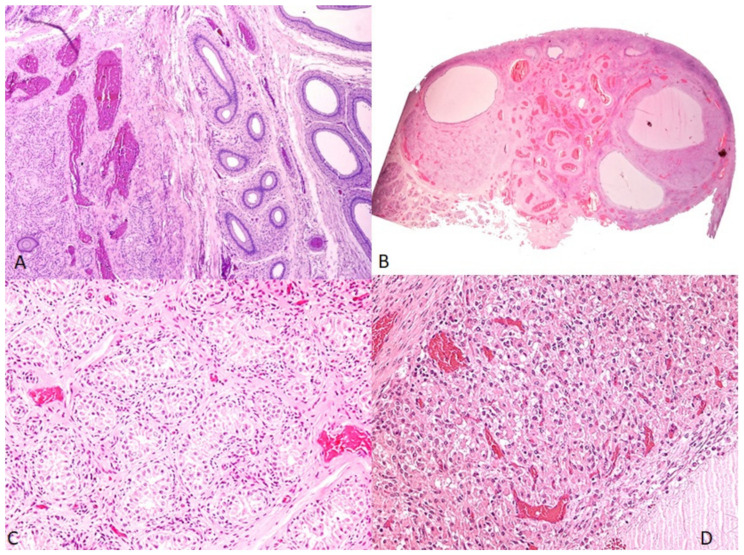
(**A**) Right-side gonad, testicle, with testicular and epidydimal components (H&E, 100×); (**B**) Left-side gonad, the ovotestis (H&E, 7.5×); (**C**) testicle-like structures of the ovotestis, evidencing seminiferous tubules lined by Sertoli cells (H&E, 100×); (**D**) ovarian structures of the ovotestis, showing secondary follicles and corpus luteum (H&E. 100×).

Microscopically, the gonad on the right side of the animal represented a testicle-like structure, with numerous solid seminiferous tubules filled with immature Sertoli cells and germ cells, in which no spermatozoa were observed. Psammomatous calcifications were noted in the center of the seminiferous tubules. The interstitium was abundant, with undifferentiated spindle cells. No mature Leydig cells were observed. The epididymis was present, surrounded by a tunica albuginea of dense irregular connective tissue (Figure 2A).

The smaller gonad on the left side represented an ovotestis (Figure 2B), with the testicular component containing seminiferous tubules lined by Sertoli cells. No spermatogenesis was seen within the seminiferous tubules (Figure 2C). The ovarian components present in the ovotestis were secondary follicles, corpus luteum, and corpus albicans (Figure 2D). The stroma was abundant. The uterine body and horns were of normal histological organization.

### 3.4. Molecular Study

After the PCR, the samples were separated on an agarose gel to confirm the amplifications. 

**Figure 3:**
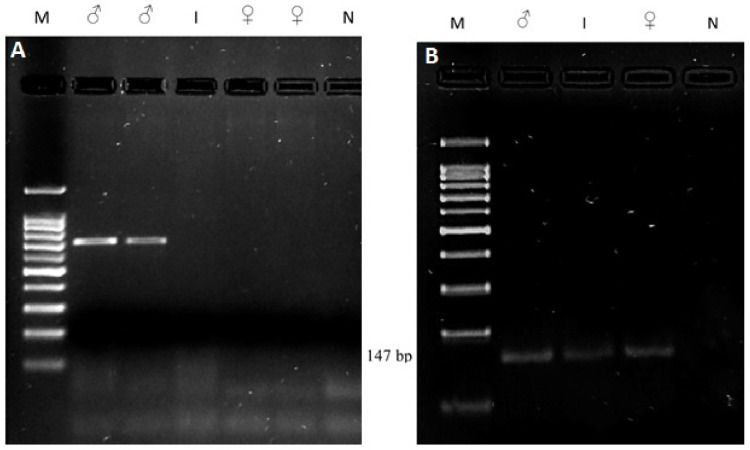
Agarose gel (1.5%) with PCR fragments. ♂ (known male roe deer); I (intersex roe deer); ♀ (known female roe deer); and N (negative control (no DNA)). A 5 μL volume of each PCR product and 1.5 μL of loading dye were deposited in each well. M: molecular marker, 100 bp Plus DNA Ladder (C: 304,105 BIORON, Germany) (**A**) *SRY* gene. A band of ~700 bp was amplified for the control males, and was subsequently sequenced. The control females did not present any amplification. The intersex roe sample did not present any band, indicating the absence of the Y chromosome (**B**) PIS mutation. All of the samples showed amplification of the expected band, thus refuting the presence of PIS deletions.

In Figure 3, a representative gel of the amplifications is presented. 

Regarding the *SRY* gene, a band was amplified for the two male controls, whereas there was no amplification of the *SRY* gene for the female controls and the intersex roe deer (Figure 3A).

Regarding the amplicon for the polled intersex syndrome (PIS) mutation, all of the samples showed amplification, indicating that the deletion was not present in these animals. 

## 4. Discussion

The existence of antlers and retained velvet is not frequent in female roe deer. In most cases, it occurs in older females with a below-average body condition [2]. By contrast, this case report was one of a young animal, estimated to be 3 to 4 years old, in good body condition. A DSD was suspected in the present case due to the simultaneous presence of the antlers, typical of a male phenotype, with a female genital phenotype. Additionally, the individual had one testis and one ovotestis. The ovary-like structures in the ovotestis were functional, in contrast to the testicle and testicle-like structures in the ovotestis. Due to the long time that had passed between the examination and sampling, the karyotype of the individual could not be determined, once it was not possible to collect any tissue or uncoagulated blood for cell culture techniques or for hormonal measurements. Blood was collected from two healthy male and two healthy female roe deer; these were used as positive controls in all of the PCRs to confirm the results obtained.

The molecular study confirmed that the roe deer specimen in the present study did not present the Y chromosome, since the *SRY* gene was not amplified for the case sample, strongly suggesting an SRY-negative, XX-chromosome constitution. Since the amplicon obtained for the *SRY* PCR amplification in the control male samples originated a band longer than expected [18], this amplicon was sequenced, allowing us to interpret a 676 bp sequence. A BLAST search was performed, showing a similarity of 100% with the *Capreolus capreolus SRY* gene (Sequence ID: HG326979.1, between nucleotide 98 and nucleotide 773). A multiple alignment (data not shown) was performed including this sequence, our own *SRY* sequence, the *SRY* sequence published by [17] (*Ovis aries* AY604733, with 528 bp), and Z30265 (*Ovis aries SRY* gene with 723 bp), considering the primers proposed by [18]. This result confirms that the amplicon obtained is from the roe deer *SRY* gene. Based on this alignment, we concluded that the 528 bp referred by [17] should consider only the sequencing result, and not the amplification result. The total length of the expected amplicon using these primers in *Ovis aries* is 695 bp, and in *Capreolus capreolus*, is 692 bp. In addition, we detected that the forward primer has one nucleotide difference, and the reverse primer has two base pair differences considering the *C. capreolus* sequence, although they hybridize and allow the amplification of *SRY* gene. Additionally, PIS deletion was not detected in this case.

Pajares et al. [9] described, for the first time, a very similar roe deer DSD case, and presented arguments based on molecular studies because, as in our work, karyotyping was not possible. In their work, the molecular approach included *SRY* amplification and a PIS-deletion test. Similarly to our case, the DSD roe deer was considered XX due to a negative *SRY* result.

Using a whole-genome sequencing approach, Kropatsch et al. [10] identified 42 genetic variations in several genes shown to be involved in sex determination (*AR*, *DMRT1*, *FGF9*, *FOXL2*, *RSPO1*, *SOX3*, *SOX9*, *SOX10,* and *Wt1*), but none were clearly established to be responsible for the pathological condition. Nevertheless, these authors confirmed, by quantitative real-time PCR analyses, a triple dose of the *SOX9* gene, allowing insights into a new genetic defect in a DSD roe deer. 

The histopathological analysis indicated that this roe deer was an XX ovotesticular DSD. The examination of the internal genitalia confirmed both female and male genital organs. The animal presented a uterine body with two uterine horns and two gonads: a testicle and an ovotestis, containing both ovarian and testicular structures. The combined presence of these structures could support the presence of the male secondary sexual features, as do the well-developed antlers in a female’s external appearance. The antler’s annual cycle is under the control of testosterone [3,9]. Another important finding was the bone antler structure revealed by CT, which highlighted similarities with that of a 2-year-old male.

DSDs are infrequent conditions in which female and male gonads can be simultaneously present, or in which the gonads contain both ovarian and testicular tissues [18]. These disorders originate from more than one genetic mechanism. The most understandable are chimeras or zygotic mosaics. The term chimera is used when two zygotes spontaneously fuse and give rise to a single embryo, but with two cell lines; cells containing XX sex chromosomes, and cells that have XY chromosomes [19]. The confirmation of this hypothesis was not possible because the lack of fresh blood prevented testing for lymphocytic chimerism. The other sex-reversal hypothesis is the presence of alterations in multiple genes involved in sexual determinism. 

DSDs are an increasing concern in both captive and free-ranging wildlife species [8]. The present case was diagnosed as an *SRY*-negative, XX ovotesticular DSD; however, the specific reason for the occurrence of this case remains unknown. A case of DSD in roe deer was described by Pajares et al. [9], but in contrast to the present case, the ovary and testicle were two independent anatomical structures. Therefore, although the present work represents the third case reported in the scientific literature for a well-studied DSD, it is the first report of a case with an ovotestis and testicle in a female-phenotype roe deer that developed growing antlers with retained velvet. 

## 5. Conclusions

The present case report confirms a DSD condition in a roe deer, and emphasizes the importance of describing these cases of DSD in mammals in order to gain a more comprehensive picture of sexual determination. 

## Figures and Tables

**Table 1:** Sequences of primers used in the PCRs with expected results.

Amplicon	Primer Sequence	Expected	Reference
*SRY*	F: 5′-ctgctatgttcagagtattg-3′ R: 5′-tcaatattgaacataagcgc-3′	528 bp fragment, if male	[17]
*PIS*	F: 5′-ttccactgcttttggtgtgt-3′ R: 5′-aacaagagaggtgccctgaa-3′	147 bp fragment: PIS-negative No amplification: PIS-positive	[13]

## Data Availability

The data that support the findings of the study are available from the corresponding author (A.M.-B.), upon reasonable request.
